# Correction: Adsorption of polycyclic aromatic hydrocarbons over CuZnFeAl–LDH modified by sodium dodecyl sulfate

**DOI:** 10.1039/d2ra90110b

**Published:** 2022-11-08

**Authors:** Boqing Liu, Jingjing Cao, Yong Jiang, Shichang Yan, Haiming He, Yu Shi, Songsong Xu, Jinhua Liang, Xiaoqian Ren

**Affiliations:** School of Chemical Engineering, Nanjing Tech University China; School of Environmental Science, Nanjing Xiaozhuang University China; School of Biotechnology and Pharmaceutical Engineering, Nanjing Tech University Nanjing Jiangsu Province 211816 China

## Abstract

Correction for ‘Adsorption of polycyclic aromatic hydrocarbons over CuZnFeAl–LDH modified by sodium dodecyl sulfate’ by Boqing Liu *et al.*, *RSC Adv.*, 2022, **12**, 25623–25632, https://doi.org/10.1039/D2RA03968K.

The authors regret that affiliations a and c were incorrectly shown in the original manuscript. The corrected list of affiliations is as shown below.

The Royal Society of Chemistry apologises for these errors and any consequent inconvenience to authors and readers.

## Supplementary Material

